# Analysis and Insights for Myths Circulating on Twitter During the COVID-19 Pandemic

**DOI:** 10.1109/OJCS.2020.3028573

**Published:** 2020-10-05

**Authors:** Shuiqiao Yang, Jiaojiao Jiang, Arindam Pal, Kun Yu, Fang Chen, Shui Yu

**Affiliations:** 1 ^1^ Data Science InstituteUniversity of Technology Sydney 1994 Sydney NSW 2007 Australia; 2 ^2^ School of Computer Science, and EngineeringUniversity of New South Wales 7800 Sydney NSW 2052 Australia; 3 ^3^ Data61CSIRO, and Cyber Security CRC Sydney NSW 2122 Australia; 4 ^4^ School of Computer ScienceUniversity of Technology Sydney 1994 Sydney NSW 2007 Australia

**Keywords:** COVID-19, myth, tweet, diffusion, emotion

## Abstract

The current COVID-19 pandemic and its uncertainty have given rise to various myths and rumours. These myths spread incredibly fast through social media, which has caused massive panic in society. In this paper, we comprehensively examined the prevailing myths related to COVID-19 in regard to the diffusion of myths, people's engagement with myths and people's subjective emotions to myths. First, we classified the myths into five categories: spread of infection, preventive measures, detection measures, treatment and miscellaneous. We collected the tweets about each category of myths from 1 January to 7 July in the year 2020. We found that the vast majority of the myth tweets were about the spread of the infection. Next, we fitted myths spreading with the *SIR* epidemic model and calculated the *basic reproduction number* $R_0$ for each category of myths. We observed that the myths about the spread of infection and preventive measures propagated faster than other categories of myths, and more miscellaneous myths raised and quickly spread from later June 2020. We further analyzed people's emotions evoked by each category of myths and found that fear was the strongest emotion in all categories of myths and around 64% of the collected tweets expressed the emotion of fear. The study in this paper provides insights for authorities and governments to understand the myths during the eruption of the pandemic, and hence enable targeted and feasible measures to demystify the most concerned myths in due time.

## I. Introduction

**Figure 1.**
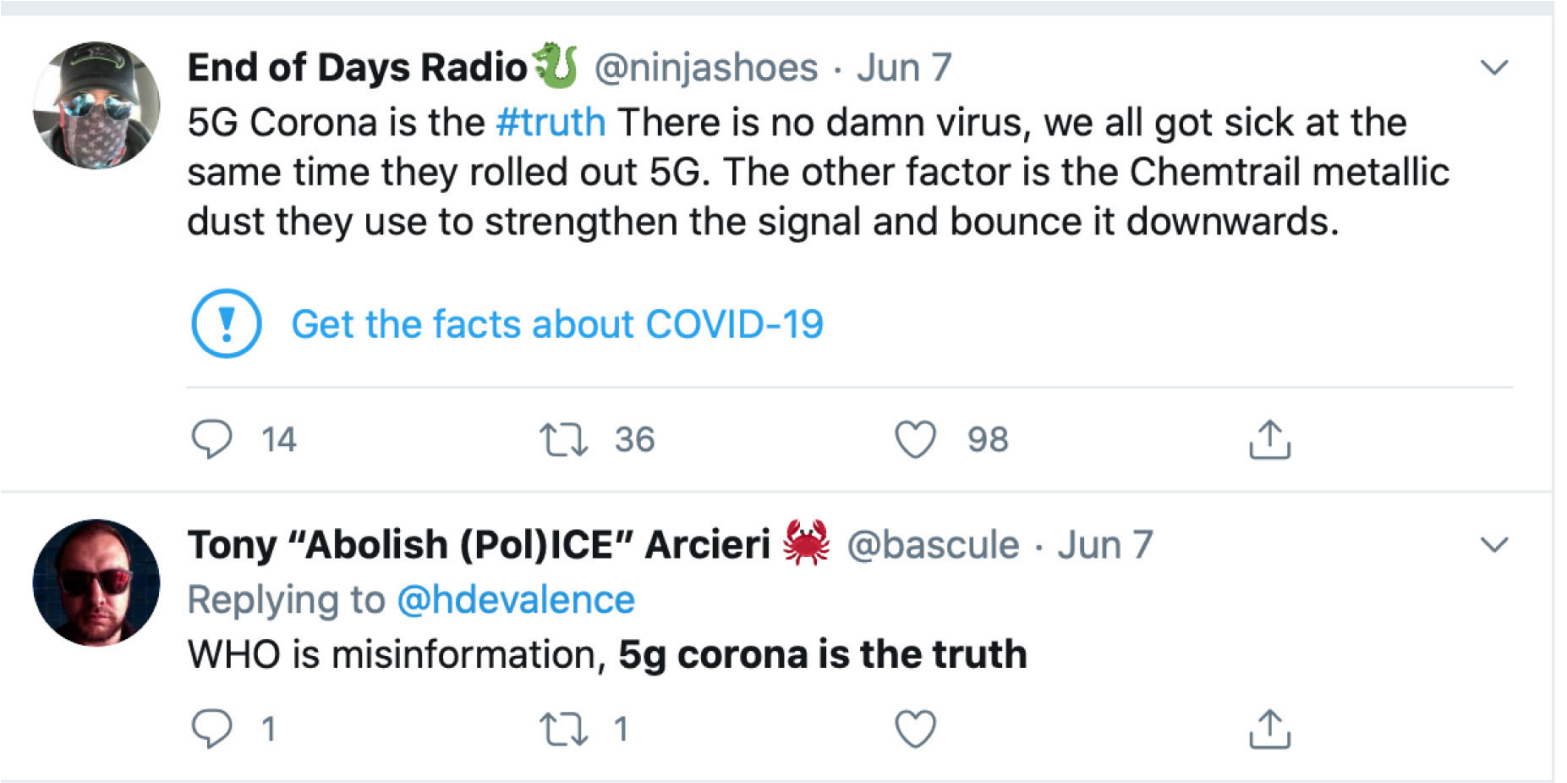
Example tweets about COVID-19 myths on Twitter.

Myths have been widely prevalent with various common infections from time to time, including Tuberculosis [1], Leprosy [2], and Flu [3]. In recent months, the world is facing COVID-19 infection, which has created havoc in the entire world and has caused severe impacts on economy, society and people's daily lives. Although authorities and governments are creating awareness and providing adequate information to the public in a timely manner, there are still a large number of myths associated with various aspects of COVID-19 infection. For example, in early April 2020, a conspiracy theory claimed 5G can spread the *coronavirus* [4]. This myth spread across the UK, and caused physical damages to mobile phone masts in Birmingham, England, even if the World Health Organisation (WHO) has clarified: “viruses cannot travel on radio waves/mobile networks”. Fig. 1 gives two example tweets about this myth. Another example myth, being shared tens of thousands of times on Facebook, alleges that garlic can prevent infection from COVID-19 [5]. These myths spread fast and extensively across the globe, particularly through social media like Twitter and Facebook.

The public has been bombarded with a vast amount of myth posts about the novel coronavirus. To fight against myths, the WHO created a series of Mythbusters^1^^1^https://www.who.int/emergencies/diseases/novel-coronavirus-2019/advice-for-public/myth-busters. based on the latest clinical and research information about the novel coronavirus. At the time of writing, there are in total 27 widely spread myths. Millions of myth posts are spreading quickly on various social media platforms. “We're not just fighting an epidemic; we're fighting an *infodemic*,” said Tedros Adhanom Ghebreyesus, Director-General of the WHO. An *infodemic* is defined as “an over-abundance of information – some accurate and some not” by the WHO. *Myth* is one of the main factors causing infodemics, which makes it hard for people to find reliable guidance and trustworthy sources. Moreover, it has been found that the pandemic of social media panic travels faster than the COVID-19 outbreak and creates mass panic and causes damaging and devastating consequences to people's daily lives [6].

Some primary research has been done by using machine learning techniques to analyze the risks of infodemics and the emotions and sentiments of the public in response to COVID-19 pandemic. For example, Galotti *et al.* [7] analyzed Twitter posts across 64 languages. They developed an Infodemic Risk Index, to quantify the rate at which a user from a country or region is exposed to unreliable posts. They found that, in low-risk regions, the level of infodemic risk remains small apart from an isolated spike in the early phase, but in high-risk regions, the infodemics are in full swing throughout the infection period. Cinelli *et al.* [8] compared people's engagement with COVID-19 memes across various social media platforms: Gab, Reddit, Instagram, Twitter, and YouTube. Kleinberg *et al.* [9] introduced the first ground truth dataset of emotional responses to COVID-19 in text form. Li *et al.* [10] compared the emotions and sentiments of the Chinese people and the American people in the time of COVID-19 based on Weibo and Twitter posts. Stella *et al.* [11] analyzed the emotional and social repercussions during the lockdown in Italy, the first country to react to the COVID-19 threat with a nationwide lockdown. They found the emergence of complex emotions, where fear and anger coexisted with solidarity, trust and hope. Yin *et al.* [12] investigated people's sentiment polarities against different COVID-19 related topics such as lockdown, stay at home, death, etc. They found that people are generally positive towards various topics but show overwhelming negative sentiment when talking about people's death due to COVID-19.

In this paper, rather than analyzing the general infodemics about COVID-19 investigated by many other papers, we focus on COVID-19 myths. We conduct a comprehensive study on the dynamics of myths diffusion, people's engagement with the myths, and people's emotions evoked by the myths. We particularly focus on the prevailing myths listed on *WHO Mythbuster*, and use the keywords of each myth to collect data from Twitter. According to the myth categorization in the letter to the editor of Asian Journal of Psychiatry [13], we separate the myths into 5 categories: the spread of infection, preventive measures, detection measures, treatments, and miscellaneous. We first analyze the most discussed topics in each category of myths. The analytical results show that people engage more in the spread of infection and the preventive measures, which provides an insight into people's top concerns in the COVID-19 pandemic. We then model the spread of myth tweets with the SIR epidemic model, which characterizes the basic reproduction number ($R_0$), where $R_0 > 1$ indicates the possibility of an infodemic [14]. The analytical results provide an assessment of the discourse evolution over time for each category of myths that an “infectious” Twitter user will create. We further analyze the account meta-data features of the Twitter users and their engagements in each category of myths, which provides an insight into people's interaction pattern in COVID-19 myths. We finally analyze the emotions evoked by each category of myths. The analytical results show that the strongest emotion evoked in the myth tweets is *fear*. Moreover, there are about 64% of tweets present fear about the COVID-19 pandemic. The comprehensive analytical results in this paper would potentially help policy-makers better understand people's concerns and thus make optimal policy.

The paper is organised as follows. We briefly go through the related work in Section II. In Section III, we describe the data and methodology used in this paper. We analyse the results and discuss the ramifications of COVID-19 myths propagation in Section IV. We conclude the remarks of this paper in Section V.

## II. Related Work

### A. Risks of Infodemic in Response to COVID-19 Pandemic

Vast infodemics have been generated around the world during the COVID-19 pandemic, which makes people difficult to discriminate the veracious information from the false. Here, we briefly review recent works in predicting infodemics and measuring people's engagement in infodemics.

To forecast potential infodemics, Galotti *et al.* [7] developed an Infodemic Risk Index (IRI) to find early warning signals of infodemics. They collected around 112 million tweets in 64 languages between 24 January 2020 and 10 March 2020. They distinguish the involved users into four classes: verified bots, unverified bots, verified humans and unverified humans. They first define the exposure $E_i$ of class $i$ through multiplying the number of followers and the number of tweets posted by the users in class $i$. Then, they define the reliability $r_m$ of a single tweet $m$ as either 0 or 1 according to the reliability of its source (fact-checked web domains). Finally, they calculate the rate (i.e., IRI) at which a user is exposed to unreliable news as the sum of the probabilities of the user exposed to unreliable tweets from all classes. They found that, in low-risk countries (*e.g.*, South Korea), the level of infodemic risk remains small apart from an isolated spike in the early phase. In high-risk countries (*e.g.*, Venezuela), the infodemics is in full swing throughout the period of observation.

To measure people's interaction and engagement with COVID-19 infodemics, Cinelli *et al.* [8] model the spread of information with epidemic propagation models and compare the information diffusion speed on different social media platforms. They collected around 8 million posts from five popular social media platforms: Gab, Reddit, Instagram, YouTube and Twitter, between 1 January 2020 and 14 February 2020. They first calculate the cumulative number of posts and the number of reactions (*e.g.*, replies, likes etc.) to these posts. Then, they adopt both the phenomenological model (*i.e.* EXP model) of [15] and the classic standard SIR (Susceptible, Infected, Recovered) compartmental model [16], to estimate the basic reproduction number $R_0$ [14] for the infodemics on each platform. They found that, all the values of $R_0$ are much greater than 1, which signals the possibility of infodemics on each of these platforms. They also noticed that people's interaction and engagement are more intense on Instagram, Twitter and YouTube than on Gab and Reddit.

### B. Public Emotions Towards the COVID-19 Pandemic

The vast infodemics overwhelm the public with a deluge of often contrasting information about the COVID-19 pandemic, which can cause a tremendous impact on the mental well-being of large populations and can even change our psychology. Here, we briefly review recent works in analyzing public emotions towards the COVID-19 pandemic.

For months, almost every newspaper has stories about the coronavirus pandemic on its front page, and this constant bombardment can cause heightened anxiety. To analyze emotions evoked by news headlines of COVID-19 outbreak, Aslam *et al.* [17] collected 141 thousand headlines carrying keyword coronavirus from top 25 English news sources from 15 January 2020 to 3 June 2020. They adopt the National Research Council Canada (NRC) Word-Emotion Lexicon [18] to calculate the presence of eight basic emotions (anger, fear, anticipation, trust, surprise, sadness, joy and disgust) and their corresponding valence in each news headline. They found that the negative emotions (*fear*, *trust*, *anticipation*, *sadness*, and *anger*) were the main emotions evoked by the news headlines. They further discussed the impact of the evoked emotions on emotional wellbeing and economic crisis, and they predicted that, in line with previous epidemic outbreaks (such as Ebola), anxiety, economic hardships, social isolation, and other similar fears will be well noted [19].

To take a close look at the public emotions and concerns from China and the United States in the time of COVID-19, Li *et al.* [10] collected 16 million posts from Weibo and 78 million English tweets from Twitter concerning the topic of COVID-19 between 20 January 2020 and 11 May 2020. They adopted the algorithm in [20] for emotion classification. More specifically, for English Twitter posts, they adopted the labelled dataset in [21] to train the classification model; for Chinese Weibo posts, they adopted the labelled dataset in [22] to train the classification model. The algorithm classifies human emotions into six categories (anger, disgust, worry, happiness, sadness, and surprise). They found significant differences in people's reactions towards COVID-19 on these two platforms. For Weibo, *worry*, *sadness* and *angry* were the most intensive emotions, and in general, the intensity of all kinds of emotions first went up steeply at the initial stage of the outbreak, staying high for about 3–5 weeks, and then gradually went down. For Twitter, the intensity of *worry*, *sadness*, and *anger* went up shortly in late January, followed by a drop. The intensities of these emotions then went up steeply in mid-March in response to the pandemic breakout in the States, reaching a peak around later March or early April, then remained steady afterwards. There is also some work focusing on analyzing the sentiment polarity of the public in the COVID-19 pandemic using social media posts. For example, Yin *et al.* [12] exploited a dynamic topic model to analysis the topic-level sentiment dynamics of people towards various COVID-19 events based on Twitter data. Zhou *et al.* [23], [24] analysed sentiment and depression dynamics for people in much fine-grained local government areas across the New South Wales state in Australia.

## III. Data and Methods

### A. COVID-19 Myths

To fight infodemics and provide answers to frequently asked questions about COVID-19, the WHO created a series Mythbusters, based on the latest clinical and research information about the novel coronavirus. Till 7th July 2020, there are in total 27 widely spread myths. The keyword(s) of each myth are listed in Table 1. We use the keywords of each myth together with a list of keywords about COVID-19 (including “covid19,” “covid-19,” “corona virus,” “coronavirus,” “2019-nCoV,” and “2019nCoV”) to actively track tweets about COVID-19 myths using Twitter API.^2^^2^https://dev.twitter.com/docs. For example, to collect the tweets about the myth of “eating garlic can prevent COVID-19,” we use the query “garlic AND (covid19 OR covid-19 OR corona virus OR coronavirus IR 2019-nCoV OR 2019nCoV)” to search tweets. The tweets used in this paper were collected from 1 January 2020 to 7 July 2020. The number of tweets and the number of distinct users involved in each myth are listed in Table 1.

**TABLE 1:** COVID-19 Myths and Their Categorization

**Type**	**Myth keyword(s)**	** **#tweets** **	**** *#users* ****	**Type**	**Myth keyword(s)**	**** *#tweets* ****	**** *#users* ****
**Prevent**	Garlic	8621	7109	**Spread**	Shoes	252	227
		Alcohol	3301		3130		Houseflies	116	111
		Hot baths	733		681		Mosquitos	22176	20062
		Rinse nose	441		400		5G Mobile networks	33236	25931
		Hot peppers	1458		1250		Cold weather, Snow	701	672
		Hand dryers	966		871		Hot and humid climates	622	598
		Bleach, Disinfectant	15926		13116		Sunny and hot weather	3969	3704
		Methanol, Ethanol	2286		2096		Older, younger people	25126	22436
		Pneumonia vaccines	2340	1935	**Treat**	Antibiotics	15068	13170
		Ultra-violet (UV) lamps	1611	1278			Medicines	5445	4407
		Chlorine	474	445	**Misc**	Recovery	531	518
**Detect**	Thermal scanners	2710	2237			Viruses, Bacteria	66	64
		Holding your breath	1224	1170			Masks, CO2 intoxication	371	326
						Masks, Exercise	133	124

Based on the myth categorization in [13] and the topic relevance of these myths, we separate the myths into five categories: Prevent, Spread, Detect, Treat, and Misc (see Table 1). More specifically, the Prevent category consists of myths of how people can prevent COVID-19, which include “eating garlic,” “drinking alcohol,” “taking a hot bath,” etc. The Spread category consists of myths of how the virus can spread among people, which includes “transmitting through houseflies,” “spreading through mosquito bites,” “spreading through 5G mobile networks,” etc. The Detect category consists of myths of how people can detect the virus, which includes “thermal scanners can detect COVID-19” and “being able to hold your breath for 10 seconds or more without coughing or feeling discomfort means you are free from COVID-19”. The Treat category consists of myths of what can treat or cure the disease, which includes “antibiotics can prevent or treat COVID-19” and “there are medicines that can prevent or treat COVID-19”. The rest of the myths constitute the Misc category, including “COVID-19 is caused by bacteria,” “the prolonged use of medical masks can cause CO2 intoxication or oxygen deficiency,” etc.

**Figure 2.**
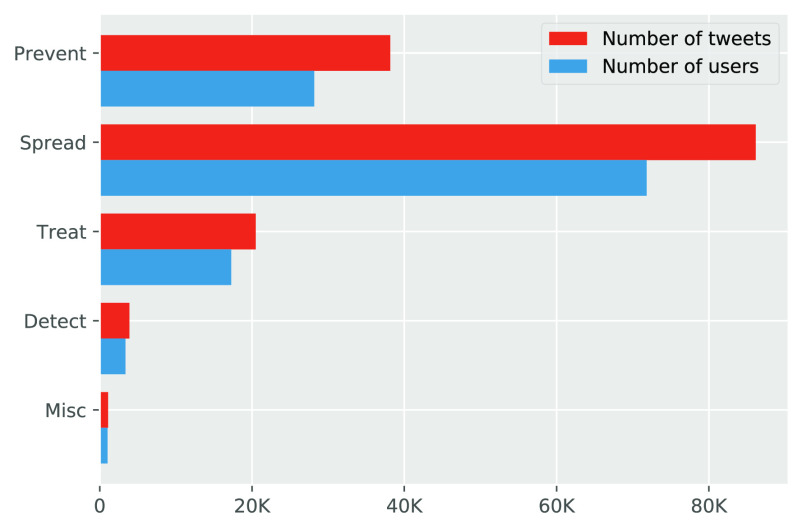
A histogram of the number of tweets and the number of distinct users across the five categories of COVID-19 myths.

From Table 1, we can see that Prevent is the major category covering 11 widely spreading myths, followed by Spread covering 8 prevalent myths, Treat covering 2 myths, Detect covering 2 myths and Misc covering 4 myths. The histogram in Fig. 2 presents the distribution of the number of tweets and the distribution of the number of distinct users across these five categories of myths. As we can see, more than 86 K tweets are about the Spread myths, which is far more than the myth tweets are about Prevent ($\approx$39 K), even though Prevent is the largest category in regard to the type of myths (covering 11 myths) while Spread is the second largest category (covering 8 myths). In other words, people expressed the biggest anxiety about how COVID-19 can spread among people followed by how we shall prevent COVID-19. Moreover, the Spread myth tweets account for more than half of all myth tweets, and the users involved in the Spread myths account for more than half of all users involved in COVID-19 myth. In fact, authorities can get a good signal from this result, that more endorsement on what can or cannot transmit COVID-19 should be advised to the general public.

### B. Modelling Information Diffusion

Many epidemic diffusion models have been adopted to simulate the propagation of information on social media. The widely used models include the Susceptible-Infected (SI) model [25], the Susceptible-Infected-Susceptible (SIS) model [26] and the Susceptible-Infected-Recovered (SIR) model [27]. In this paper, we assume that the people who get involved in myth spreading can later realize it is a myth or rumor when the authorities or governments clarify the myth or they realize it by themselves (i.e., they have *recovered*). Therefore, we employ the SIR model. The SIR model can be described by the following set of differential equations:

\begin{align*}
\frac{\partial S}{\partial t} &= -\beta SI, \tag{1}
\\
\frac{\partial I}{\partial t} &= \beta SI -\gamma I, \tag{2}
\\
\frac{\partial R}{\partial t} &= \gamma I. \tag{3}
\end{align*}

where $S$ is the fraction of susceptible individuals to be infected at time $t$, $I$ is the fraction of infectious individuals at time $t$, and $R$ is the fraction of recovered individuals at time $t$; $\beta$ is the transmission rate per infectious individual, and $\gamma$ is the recovery rate. Thus, if $\beta - \gamma > 0$, then $I(t)$ grows exponentially about the DFE (disease-free equilibrium: $S=1, I=0, R=0$). Similar to [8], we interpret the number $I + R$ as the number of distinct users that have posted a tweet on the myth.

In this paper, to further measure the speed of myth diffusion, we employ the basic reproduction number $R_0$ [14], i.e., the expected number of infections directly generated by an infected individual for a given time period. Similar to epidemics, the myth is considered to be dangerous if $R_0 > 1$, i.e., if an exponential growth in the number of infections is expected at least in the initial phase. To estimate the basic reproduction number $R_0$, we use the negative log likelihood estimates of the models’ parameters [28]. The range of parameters is estimated via the Nelder-Mead algorithm [29].

Modelling the dynamics of COVID-19 myths on social media through reproducing the real diffusion is important for risk assessment, assessing the impact of authority policies, and optimizing control and counter-measure strategies. In Section IV-B, we will analyze the modelling results of myth tweets spreading among Twitter users.

### C. Emotion Analysis

There has been a number of work on annotating texts based on emotions [30]–[32], and a number of machine learning approaches have been developed for automatic emotions detection, including bag-of-words (BoW) models [33], latent semantic indexing (LSI) models [34], and neural network models [35]. Meanwhile, various classes of discrete emotions are adopted in different approaches. For example, Mohammad *et al.* [36] created the first annotated dataset for four classes of emotions: anger, fear, joy, and sadness. The SemEval-2018 Task 1e dataset [37] labelled each tweet in the dataset with one or multiple emotion labels, where the dataset contains eleven emotion classes in total, i.e., anger, anticipation, disgust, fear, joy, love, optimism, pessimism, sadness, surprise and trust. In this paper, we adopted the well-established emotion theory by Paul Ekman *et al.* [38] which groups human emotions into six classes: anger, disgust, fear, joy, sadness, and surprise. We employ the deep neural network based model proposed by Niko *et al.* [35] for emotion classification.

The deep neural network (NN) based model proposed in [35] can use both recurrent (RNN) and convolutional (CNN) networks as the core module. The model consists of four main components. They first proposed two different levels of granularity for model input: tokenizing each tweet's content and then feeding a sequence of tokens into the NN, or passing characters of each tweet one by one into the NN. Then, the sequences of either tokens or characters were embedded as vectors by utilizing the pre-trained GloVe embedding [39]. Thirdly, the embeddings were passed through the dropout layer to prevent NN from overfitting. Fourthly, the dropout layer results were passed into either RNN or CNN layers, where dropout layers needed to be put in between layers if multiple RNN or CNN layers were used. In this paper, we directly adopt the fully trained models shared by Niko *et al.*^3^^3^https://github.com/nikicc/twitter-emotion-recognition. to predict the emotions of each tweet related to COVID-19 myths. In particular, the character level of granularity is used to process input data, and RNN is used as the core module. In Table 4, we list the predicted emotions for some example tweets of COVID-19 myths and the probabilities of each tweet presenting different classes of emotions by using this model.

**TABLE 2:** Descriptive Statistics on Users WHO Participated in Different Myth Cascades

****	**mean (log)**					**stdv (log)**				
		Prevent	Spread	Treat	Detect	Misc	Prevent	Spread	Treat	Detect	Misc
**followers**	15.12	14.70	14.30	15.67	12.52	18.78	18.52	18.55	18.86	15.02
**followees**	10.81	10.71	10.71	10.56	10.22	18.78	18.52	18.55	18.89	15.02
**verified**	2.06	2.37	2.44	2.21	0.80	2.06	2.45	2.28	2.20	0.86
**engagement**	14.74	14.58	14.60	15.14	14.44	16.15	15.98	16.11	16.46	15.98
**account age**	11.08	11.03	10.99	11.07	10.91	10.44	10.42	10.42	10.42	10.46

**TABLE 3:** Descriptive Statistics on Tweets in Myth Cascades

****	**mean**					**stdv**				
		Prevent	Spread	Treat	Detect	Misc	Prevent	Spread	Treat	Detect	Misc
**retweets**	6.19	4.33	6.11	9.35	2.45	105.08	126.08	125.93	139.46	16.67
**favorites**	17.67	13.23	14.74	27.20	5.97	340.29	506.80	301.17	443.60	33.77

**TABLE 4:** Example Tweets and the Predicted Emotion Distributions

**Tweet**	**Anger**	**Disgust**	**Fear**	**Joy**	**Sadness**	**Surprise**
“We've been waiting and still waiting for medicine for corona virus Karim and all you know or talk about is judging people who drink alcohol and they are not even kids that's what they want to do why are always telling them what to do? months of waiting and people are still dying”	0.048	0.198	**0.662**	0.010	0.044	0.038
“My whatsap media is filled up with BC and videos of corona virus related issues. In a day I'm sure of receiving atleast 10 some I've never opened self, now it's 5G own that's reigning”	0.020	0.004	**0.524**	0.420	0.006	0.025
“I don't get why old people risk getting the corona virus for lottery like I PROMISE you the 2$ you MIGHT win is not worth it, also it's nowhere near essential”	0.050	0.000	0.134	0.027	**0.785**	0.003
“Our great great Grandma's & fa's told always keep nature termeric powder, peppercorn, stone salt, garlic, ginger, lime this all very big medicine for #coronavirus #Rajinikanth #NarendraModi #DonaldTrump”	0.015	0.001	0.228	**0.752**	0.002	0.002
“I was flipping through channels and heard the President of the United States literally suggest that we could inject people with bleach to kill the corona virus. Excuse me while I go beat my head against the wall”	**0.684**	0.004	0.182	0.124	0.004	0.002

Automatically detecting emotions in social media posts about COVID-19 myths is important for authorities and governments to understand people's reactions towards the myths and thus take correct and targeted actions to dissolve people's anxieties. In Section IV-D, we will analyze the emotion patterns of people's reaction towards COVID-19 myths.

## IV. Analyses and Insights

### A. The Most Discussed COVID-19 Myth Topics

In this section, we take a close look at the most discussed topics in each category of myths. We particularly extract the most frequent terms (*i.e.* words or phrases) in each category of myths, and filter the COVID-19 keywords (including “covid19,” “covid-19,” “corona virus,” “coronavirus,” “2019-nCoV” and “2019nCoV”) out of the frequent terms so as to get a clearer view of the discussed topics. Meanwhile, we use the Porter Stemming method for surfix stripping^4^^4^https://radimrehurek.com/gensim/parsing/porter.html. and count the frequency of each term after the stemming.

**Figure 3.**
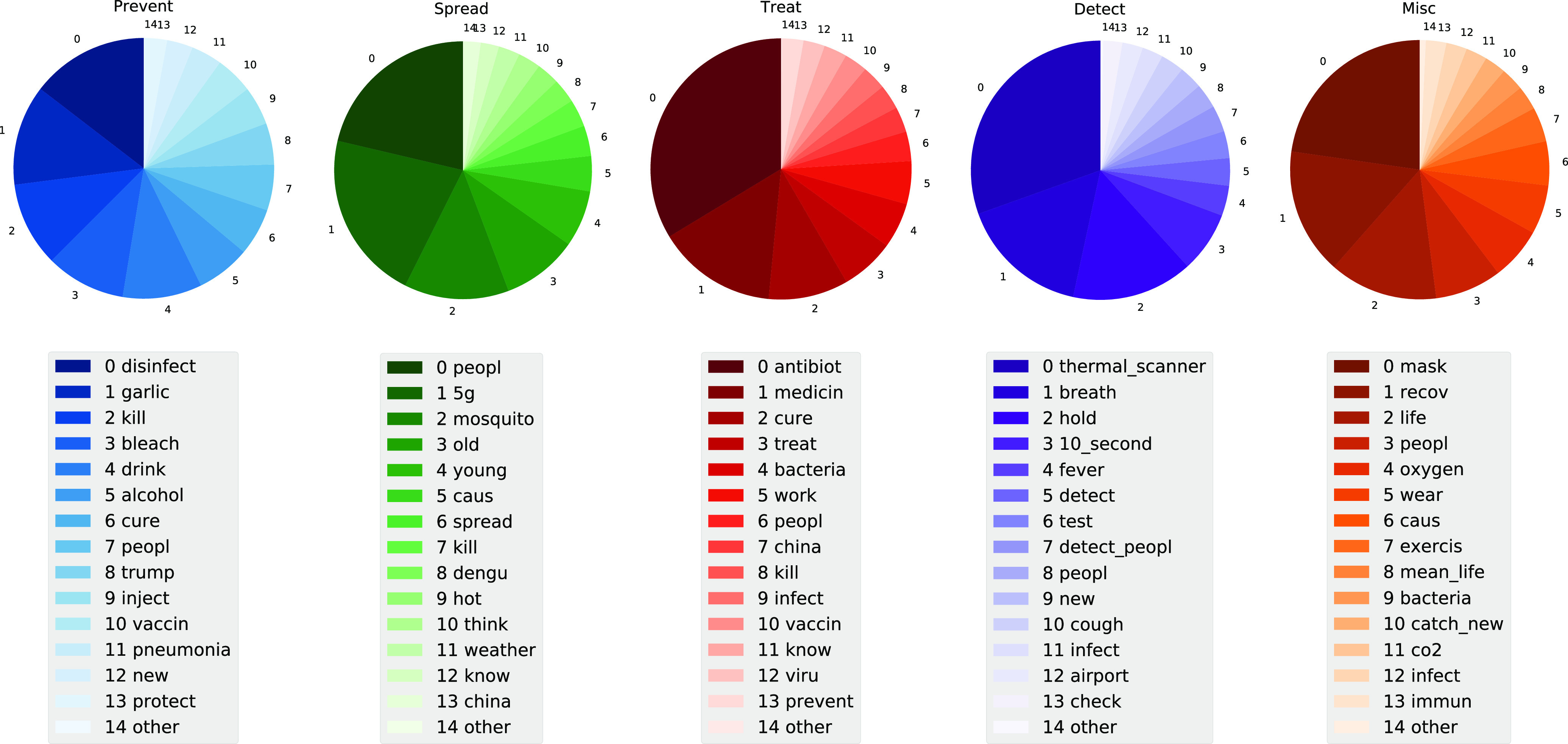
The lower panel presents the most discussed terms for each category of COVID-19 myth. The top panel presents the proportion of these terms in their corresponding category of myth as pie charts.

Fig. 3 (the lower panel) shows the top 15 frequent terms for each category of myths, where we use the term “other” to represent the remaining words in each category for simplification and data integrity. The proportions of the tweets containing these frequent terms in each category of myths are shown in Fig. 2 (the upper panel) as pie charts, where the proportion of the term “other” is computed as the proportion of those tweets not containing the top 14 frequent terms.

As we can see from Fig. 3, in the Prevent myth tweets, the most discussed myth topics are “injecting disinfectant/bleach,” “eating garlic” and “drinking alcohol” to “kill the virus” and “protect people” against COVID-19. Note that, “trump” is also listed as one of the top frequent terms, which is due to the fact that some myths (including Bleach, Disinfection and Methanol, Ethanol in Table 1) were triggered after US President Donald Trump suggested research into if coronavirus might be treated by injecting disinfectant into the body [40]. Due to his popular public effect, it brought hot discussion on social media [41]. In the Spread myth tweets, the most discussed myth topics are “5G” and “mosquito dengue” can spread the coronavirus, “coronavirus just kill old people” and “coronavirus cannot spread in hot weather”. In the Treat myth tweets, the most discussed myth topics are “antibiotics can kill the coronavirus” and “there is specific medicine to treat/cure the coronavirus”. In the Detect myth tweets, the myth topics “thermal scanner” and “holding breath for 10 seconds without coughing” can detect the coronavirus are most discussed. In the Misc myth tweets, the myths about “wearing masks,” “recovering from coronavirus” are most discussed.

**Figure 4.**
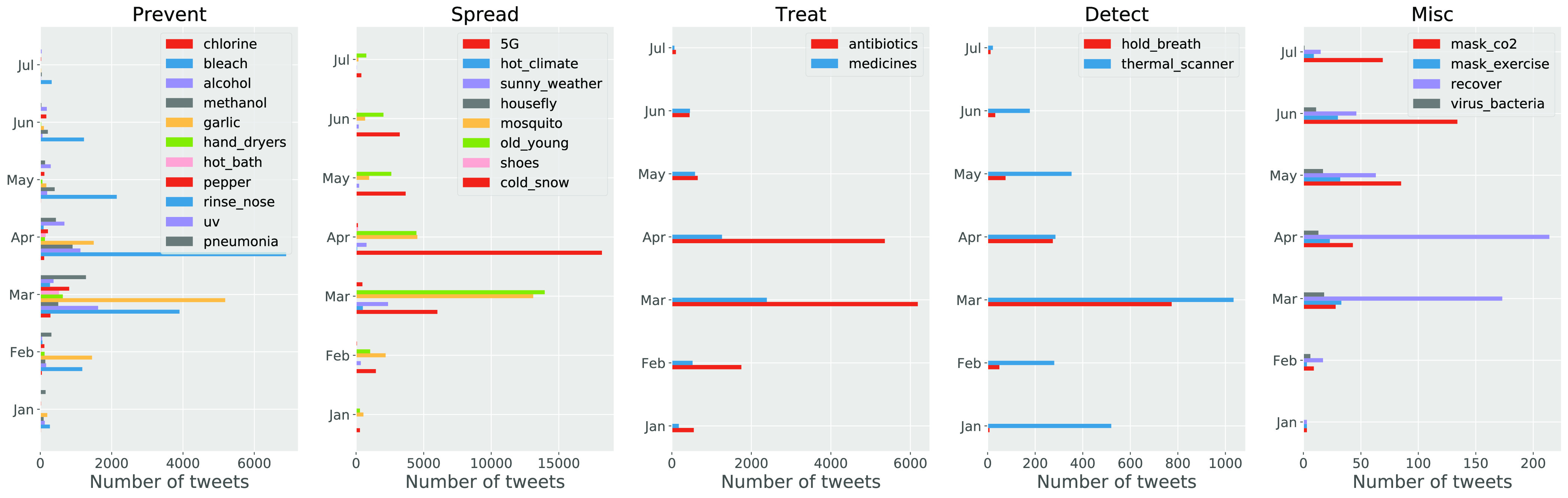
The dynamics of the number of myth tweets in each category of myths from 1 January 2020 to 7 July 2020.

Fig. 4 shows the dynamics of the number of tweets of each myth from January to early July in 2020. We see that most myths started to spread from February, they became prevalent from March and spread even broader in April, the spreading speed slowed down from May, and gradually fewer and fewer tweets were posted in June and early July. Note that, the WHO launched the Mythbusters to dispel myths from early February, but due to the global spreading of COVID-19 (WHO characterized COVID-19 as a pandemic on 11 March 2020), more myths were generated and a massive number of users and social media posts were involved in myths propagation from March. A good signal can be observed from this figure is that a decreasing number of myth tweets were posted from May. This is due to the fact that authorities, governments and social media companies (like Facebook, Google, Pinterest, Tencent, Twitter, TikTok, YouTube and others) started to put more efforts on countering the spread of myths. For example, Twitter adds fact-checking labels on those tweets that link myths and rumours with the coronavirus.^5^^5^https://www.cnbc.com/2020/06/08/twitter-5g-coronavirus.html. However, as we can see in the panel of Misc myths in Fig. 4, new myths (*e.g.* “mask_co2”) are continuously generated with the continuous spread of COVID-19. This indicates that myths will always exist with the infection, so that authorities, governments and social media companies will have to dispel myths during the whole time of the infection.

### B. The Diffusion of COVID-19 Myth on Twitter

**Figure 5.**
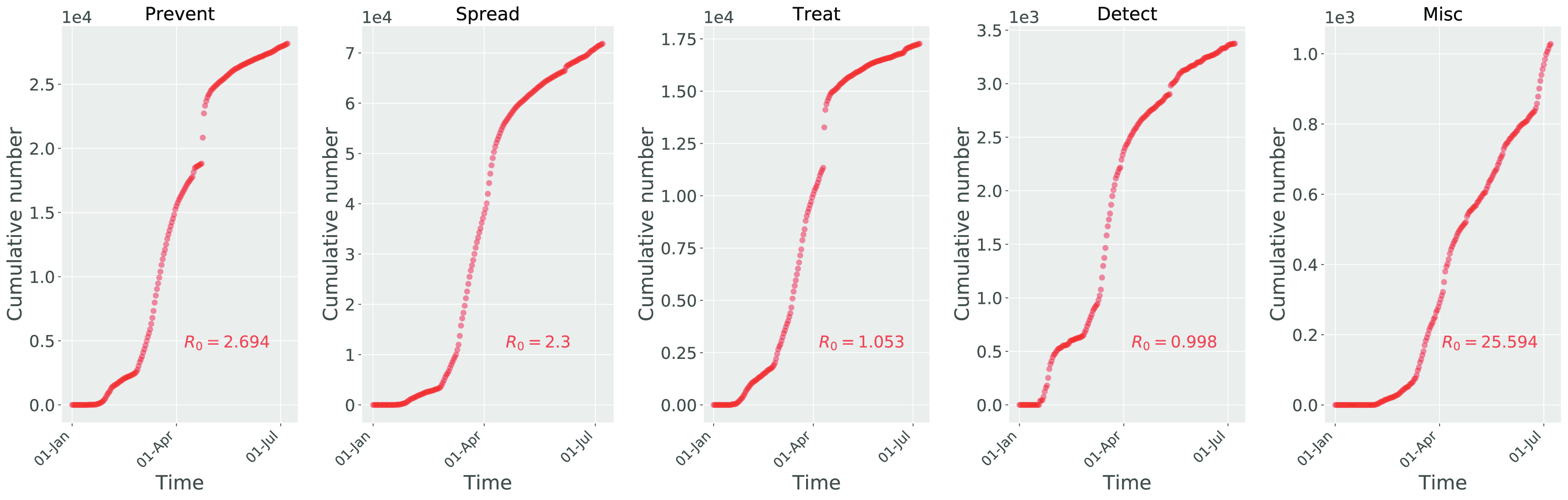
Accumulative user number distribution for each category of myth.

In this section, we model the diffusion of COVID-19 myths so as to estimate the diffusion trend of each category of myths and compare their diffusion speed on Twitter. We adopt the SIR model introduced in Section III-B to simulate the diffusion of tweets about each category of myths. Fig. 5 shows the cumulative number of new tweets about each category of COVID-19 myths each day. As we can see, in general, all categories of myths spread fast on Twitter, and they started to attract massive attention in March. The basic reproduction number $R_0$ is calculated for each category of myths (see Fig. 5). As we can see all categories of myths developed an infodemic (with $R_0 > 1$) except the myth category of Detect (with $R_0=0.998$). This indicates that people worry much more about the spread of the infection, the preventive measures, and treatment of the coronavirus, while worrying less on the detection measures of the coronavirus. Note that, the myth category of Misc presents a much greater reproduction number (with $R_0=25.594$) than other myth categories, which is due to a sudden increase of the number of myth tweets at the end of the observation duration where many users were newly “infected” and have not got “recovered”. This is consistent with the result in the Misc panel of Fig. 4.

As we can in Fig. 5, the Prevent myth tweets increase sharply during March and April, and the increase leaped at the end of April before slowing down the increase speed shortly after the leap. The Spread myth tweets increase sharply during March, and the increase leaped in early April before slowing down the increase speed shortly after the leap. Similarly, the Treat myth tweets increase sharply during March, and the increase leaped in early April before slowing down the increase speed shortly after the leap. The Detect myth tweets increase sharply during March, but the increase speed slowed down from April. The Misc myth tweets increase steady from March, and the increase leaped in early July without the leap.

### C. People and Their Engagement in COVID-19 Myths

In this section, we take a close look at the users involved in spreading COVID-19 myths and their engagement in these myths. We first analyze the meta-data features of the users involved in each category of myths, including their average follower count, their average followee count, the average number of verified users, their average engagement, and their average account age. The statistical results are shown in Table 2, where we calculate the log of each value to avoid large numbers. As we can see in Table 2, there is not a significant difference in users’ meta-data features across different categories of myths, except the Misc category. For example, in terms of “followers count,” the users involved in the first four categories of myths present similar mean “followers count” around 14 to 15, while the Misc category users present a lower mean “followers count”. Similarly, in terms of the feature “followees count,” the users involved in the first four categories of myths present similar mean “followees count” around 10.5 to 10.8, while the Misc category users present a lower mean “followees count”. A noticeable difference is the number of verified users involved in these five categories of myths. As we can see, for the users who have been involved in the Misc myths, fewer of their Twitter accounts are verified, while for the users involved in the first categories of myths, many more of their Twitter accounts are verified. Similarly, the users involved in Misc myths present lower engagement and younger account age than users involved in other categories of myths. These meta-data features are often used for social bots detection [42]. In particular, those accounts that are not verified, and with younger account age and lower engagement are often detected as bots. Therefore, the results in Table 2 indicate that more bots are used in spreading Misc (or new) myths.

Then, we analyze people's reactions to different categories of myths. We particularly use the average retweet count and favorite count of the tweets in a myth category to measure people's reaction to the myths in the category. The statistical results are shown in Table 3. As we can see, people show the greatest intensive reactions towards the Detect myth tweets, followed by Prevent, Treat, and Spread myth tweets, and people show the lowest intensive reactions towards the Misc myth tweets.

**Figure 6.**
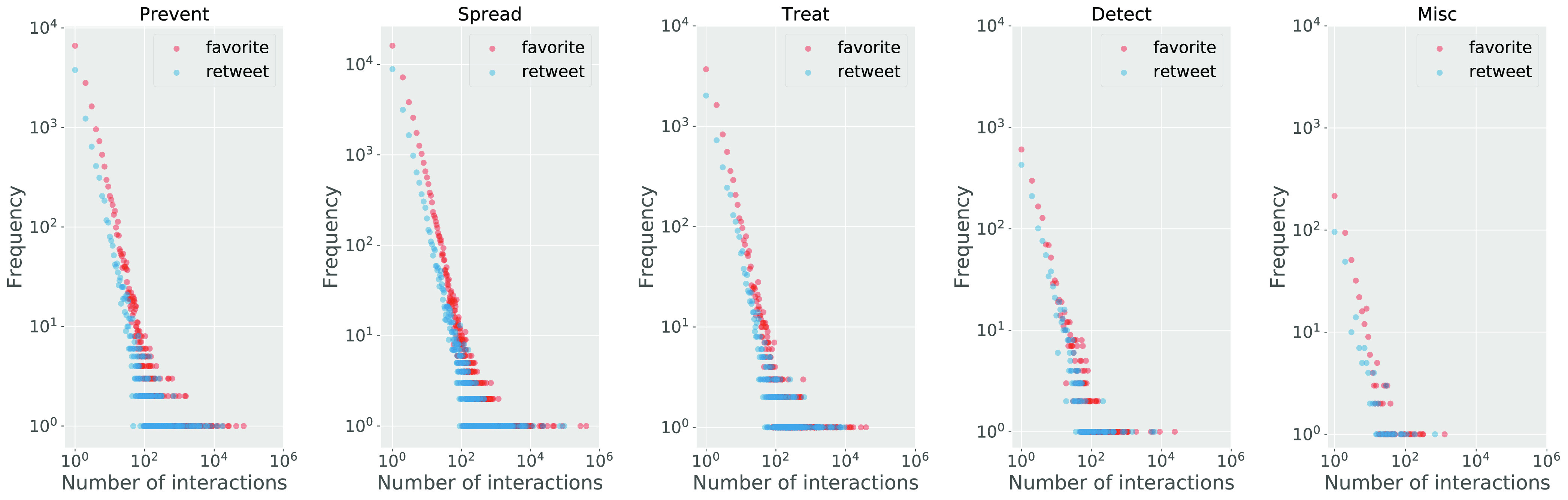
The distribution of people's engagement (retweet count and favorite count) in each category of myths.

Finally, we analyze the distribution of people's engagement in each category of myths. The statistical results are shown in Fig. 6. The highest volume of reactions in terms of retweets and favorites can be observed on mainstream myths such as Prevent and Spread. Despite the differences among the myth categories, we observe that they all display a rather similar distribution of the users’ engagement characterized by a long tail. Indeed, users’ reactions towards the COVID-19 myths present attention patterns similar to any other topic [43].

### D. People's Emotions Towards COVID-19 Myths

In this section, we adopt the trained deep neural network model [35] introduced in Section III-C to analyze people's emotion toward different categories of COVID-19 myths. The model was trained on the labelled datasets which contain six different kinds of emotions (i.e., *anger, disgust, fear, joy, sadness, surprise*). For each tweet, the model could predict its emotions with the associated probability. The detailed structure of the model can be found in [35]. Based on the trained model, we can predict the emotions for our collected myth tweets. Table 4 shows, for five example tweets, the emotion distribution estimated by the deep neural network model. Taking the first example tweet as an example, the model estimates that this tweet is 0.662 of fear, is 0.198 of disgust, is 0.048 of anger, etc. Hence, fear is the strongest emotion expressed by this tweet.

**Figure 7.**
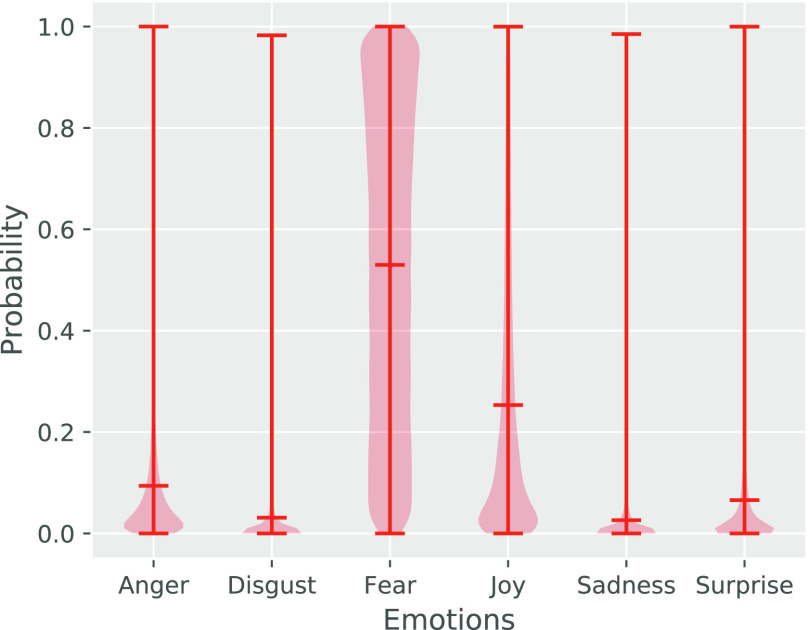
Emotion distributions across all of the tweets.

To quantify the overall emotion distribution across all of the collected tweets, we show in Fig. 7 the distributions (given as a *violin plot*) of each of the six classes of emotions. Taking distribution of the emotion “anger” as an example, the probability of “anger” expressed in all myth tweets ranges from 0.0 to 1.0, with a majority of the probabilities located between 0.0 and 0.12, and the mean probability is 0.09. As we can see from Fig. 7, the highest predictive emotion class is “fear” (mean probability 0.55), followed by “Joy” (mean probability 0.25), and “anger” (mean probability 0.09), while “surprise,” “sadness” and “disgust” are the least probable emotions in the myth tweets. This is closely related to the myths about the coronavirus *prevention* and *spreading*.

For example, for the myths in the Prevent category, there exist myths such as injecting disinfectant and exposing to ultraviolet light to kill the virus. These myths may terrify ordinary human beings who want to keep safe but do not have related background knowledge for the COVID-19. Interestingly, the second highest predicted emotion is “joy”. More specifically, every myth tweet averagely present about 25% of “joy”. This may because that some myths are absurd and people may tweet/retweet these myths in a humorous way. For instance, many people post funny tweets commenting the myth that “being able to hold your breath for 10 seconds or more without coughing or feeling discomfort means you are free from COVID-19” and “5G mobile network can spread virus”. This, in another way, indicates that many people can recognize those myths, but they generally express fear in their posts. The third highest predicted emotion is “anger,” that every myth tweet present about 10% of “anger,” followed by surprise, disgust and sadness.

**Figure 8.**
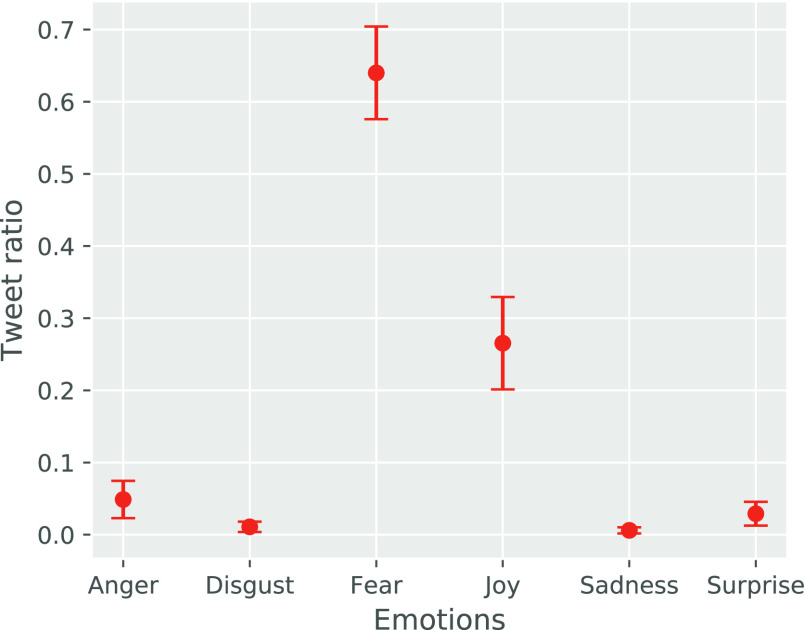
The proportion of tweets across each kind of emotion.

The *box plot* given in Fig. 8 shows the ratio of tweets for each class of emotion. More specifically, for an arbitrary tweet, we use the emotion with the largest probability as the emotion of the tweet. Taking the first tweet in Table 4 as an example, “fear” is the emotion with the largest probability, so we use “fear” to represent the emotion of this tweet. As we can see in Fig. 8 that about 64% of tweets are discussing “fear” and about 27% of tweets are discussing about “joy”. This is consistent with the results in Fig. 7.

### E. Summary

From the above results, it is clear that people are mostly worried about the *spreading* of coronavirus. They think that it's spreading through the *5G mobile networks*. Since the $R_0$ value for spreading is quite high (2.3), the myths are propagating rapidly. The most common emotion among people is *fear*. This insight is consistent with what is reported in the print, digital, online, and social media. During the initial phase of a pandemic like COVID-19, it is expected that people will be scared about how the virus is spreading rapidly in the general population. The surprising finding is that many people wrongly believe that the virus can spread through mobile networks. They are mistakenly identifying a *biological virus* with a *computer virus*.

Another common misconception is that *drinking alcohol* can *prevent* the infection of coronavirus. Since drinking of alcohol is associated with joy and pleasure, this makes people feel relaxed and reduces their stress. Since the $R_0$ value for prevent is very high (2.69), these myths are also propagating pretty fast. While this is not entirely unexpected, the fact that many people believe that consuming alcohol will protect them against COVID-19 is an eye-opening revelation of our study.

## V. Conclusion

In this paper, we comprehensively analyzed the widely spread myths about the COVID-19 pandemic. We particularly analyzed the most discussed topics in each category of myths, modelled the dynamics of myths diffusion, people's engagement with the myths, and people's emotions towards the myths. The analytical results indicate that the myths about the spread of COVID-19 and the preventive measures of COVID-19 attract more attention from people. The reasons behind the phenomena may be closely related to the facts that the number of globally confirmed COVID-19 cases increases rapidly during the studied period. Thus, the myths about pandemic prevention or spreading can easily catch people's attention and mislead people to the incorrect understanding of COVID-19. Moreover, the strongest emotion evoked in the myth tweets was fear and there are about 64% of tweets present fear about the COVID-19 pandemic. The comprehensive analytical results in this paper would potentially help policy-makers better understand people's concerns and thus make optimal policy.
